# Effects of Reference Group Instructions on Big Five Trait Scores

**DOI:** 10.1177/10731911231175850

**Published:** 2023-05-29

**Authors:** Madeline R. Lenhausen, Wiebke Bleidorn, Christopher J. Hopwood

**Affiliations:** 1 University of California, Davis, USA; 2 University of Zurich, Switzerland

**Keywords:** Big Five, personality assessment, traits, reference group effects, gender, validity

## Abstract

People responding to personality questionnaires rate themselves by comparing themselves to some reference group, but this reference group is typically not specified. In this study, we examined the differences between Big Five trait scores when people responded to trait questionnaires without a specified reference group, as is typical in personality assessment, and when they were asked to compare themselves to people in general, close others, people their age, people their same gender, their ideal self, or their past self. We found that personality scores tended to be more adaptive for between-person comparisons than for within-person comparisons. We also found that unprompted instructions produced mildly higher scores across all traits. There were few differences among between-person reference group conditions. Men rated themselves as slightly more agreeable when comparing themselves to other men. Implications for basic and applied personality assessment are discussed.

Responding to personality questionnaire items is a complex cognitive process (T. B. Rogers, 1974), and people are likely to vary in how they approach this task (A. M. Wood et al., 2012). Systematic individual differences in these processes may be confounded with valid variance in personality test scores. One potential source of systematic nontrait variance is the reference group against which people compare themselves when rating items. For instance, a psychotherapy patient may rate themselves as relatively higher in neuroticism than people in the general population but similar in neuroticism compared with other psychotherapy patients, based on a correct inference that people in psychotherapy tend to be more neurotic, on average. This variation in comparison has been referred to as the reference group effect (RGE; Heine et al., 2002). Typically, personality questionnaires are administered with vague instructions about what reference group to use (Credé et al., 2010), and thus, little is known both in individual clinical assessment and in research about the impact variation in reference groups could have on the data that results. The goal of this study was to examine how specific instructions for participants to compare themselves to reference groups affect scores on a measure of Big Five personality traits.

This study builds on a previous report using the same data (Lenhausen et al., 2022). That previous study focused on which reference group people believe they use and which reference group the data suggest they actually use. Among 1,227 respondents, 40% reported comparing themselves to “people in general” when completing a questionnaire, whereas 16% reported comparing themselves to close others, 15% to their ideal self, and 14% to people their same age. However, a comparison of data from unprompted instructions with data in which specific reference groups were prompted revealed that instructing people to compare themselves to people their same age resulted in scores that were more similar to those from the unprompted instructions than to any other reference group demand. This suggested that people typically compare themselves to people their same age when completing questionnaires, even though a plurality of people believe they are comparing themselves to people in general. In that previous study, we did not report or focus on differences in mean levels of personality traits across different reference group instructions. That is the focus of the current study.

## Relevance of RGEs in Applied Assessment

Variation in RGEs across groups can obscure group comparisons (Heine et al., 2002, 2008). For instance, mean differences between two groups might reflect substantive differences in psychological features, or it might reflect systematic differences in the reference groups chosen when completing questionnaires (Peng et al., 1997; van de Gaer et al., 2012). To the degree that RGEs are systematic, reference group processes contribute to reliable variance in personality test scores that is typically not accounted for (Guimond et al., 2007; Pulford et al., 2018) and could bias assessment data. Previous studies have found that RGEs can reduce the validity of personality assessment scores more generally (Credé et al., 2010; Frank, 2012; Marsh et al., 2020; Mõttus et al., 2012; van de Gaer et al., 2012). Thus, accounting for RGEs could reduce bias and enhance validity in psychological assessment.

A better understanding of the impact of different reference group instructions could also be exploited in clinical assessment or personality research. In clinical practice, there may be implicit demands in clinical settings for respondents to compare their personality to another aspect of themselves, such as how they were in the past or how they would like to be. For instance, during an initial assessment, patients may be focused on how they would like to be and, thus, rate their personality in terms of how they are falling short of that ideal. This would likely make their personality seem less adaptive than if they were to compare themselves to the average person, which is typically what clinicians assume as the reference group in such a situation. Conversely, the patient may assume when given a follow-up personality assessment that the implicit demand is to compare their current levels to their past. To the extent that scores would change when the patient compares their personality to some other version of themselves as opposed to other people, miscommunication between the patient and clinician about the reference could result in misunderstanding.

In basic personality research, it may be possible to specify certain reference groups in survey instructions to limit the impact of systematic RGE variation on personality scores. This could be particularly useful in cross-cultural research in which it may matter if the participant is comparing themselves to people within or beyond their own culture or in longitudinal research where there may be an implicit demand to respond based on perceptions of change. However, using RGEs to generate more specific inferences about personality assessment data in either clinical practice or basic research would require a better understanding of the impact of RGEs on personality test scores.

## Influence of Reference Group Instructions on Trait Scores

A. M. Wood et al. (2012) had participants read multiple prompts in which an individual’s behavior was held constant across conditions, whereas the behavior of the surrounding people changed. They found that individuals’ Big Five trait scores were rated differently by participants depending on the reference group’s levels of the corresponding trait. Specifically, an increase in the number of people who ranked lower than the target individual in the corresponding trait was associated with an increase in ratings of the target individual’s trait. In addition, an increase in the number of people who ranked higher than the target individual in the corresponding trait was associated with a decrease in ratings of the target individual’s trait. This study demonstrated that the explicit use of different comparison populations can affect individual trait scores.

Credé et al. (2010) collected self-reports of conscientiousness in a “reference-group-free” condition (no reference group prompted), as well as in four additional reference group conditions (immediate family, people of same age and gender, close friends and peers, people in general). They found that prompting different reference groups resulted in significant differences in a participant’s test scores. For example, instructing participants to refer to people of their same age/gender when responding to items produced higher conscientiousness scores than when instructing participants to refer to people in their immediate family.

These initial studies indicate that the use of reference groups can influence test scores. However, thus far, little is known about the effects of differential reference group selection, and more research is needed to understand how specific reference group instructions differentially affect personality trait scores.

In this preregistered study, we compared people’s Big Five trait scores across seven conditions: (a) unprompted, (b) comparisons to people in general, (c) comparisons to people the respondent knows well, (d) comparisons to people the respondent’s same age, (e) comparisons to people the respondent’s same gender, (f) comparisons to how the respondent was in the past, and (g) comparisons to the respondent’s ideal self. Consistent with Credé et al (2010), we expected that prompting participants to use different reference groups when responding to personality questionnaire items would affect their personality trait scores. We expected that the “close others” reference group (e.g., romantic partner, close friends/family) would produce lower scores of conscientiousness than the remaining reference groups. This prediction aligns with findings from Credé et al. and may occur because of a general positivity bias that people have in their perceptions of people close to them. We expected the ideal self reference group to produce more maladjusted levels of all Big Five traits, that is, lower levels of agreeableness and conscientiousness and higher levels of neuroticism, because people logically think of themselves as having less adaptive personality traits than their ideal selves. Similarly, we expected that the past self reference group would produce more well-adjusted levels of the Big Five traits, that is, higher levels of agreeableness and conscientiousness and lower levels of neuroticism, because of the overall personality maturation over the lifespan as well as people’s general tendency to believe they are doing better than they were in the past (Bleidorn et al., 2022; Schwaba et al., 2022). Finally, given past research on self-stereotyping with regard to gender, we expected the same gender reference group to differentially influence agreeableness and neuroticism scores depending on the participant’s gender. Specifically, given that women tend to score higher in levels of neuroticism and agreeableness than men (Weisberg et al., 2011), we expected the same gender reference group for women to produce lower scores of agreeableness and neuroticism than their trait scores in other reference group conditions. Similarly, we expected the same gender reference group for men to produce higher scores of agreeableness and neuroticism than their trait scores in other reference group conditions. In other words, the absence of the opposite gender when making trait comparisons could dull the contrast between one’s own agreeableness and neuroticism and others’ levels of these traits. We make no specific predictions about the remaining reference groups on each of the traits but plan to explore their differences.

## Methods

### Sample

We recruited 1,227 participants with a target sample of 1,000 participants from Prolific—an online data-collection service. We asked one exclusionary item to exclude participants who were not paying attention during the study: “Please select ‘Somewhat agree’ for this question. Thank you for paying attention.” We removed data from participants who did not finish the study and/or failed the attention check (*n* = 68), giving us a total sample of *N* = 1,194, of which 51.42% were female, 46.73% male, and 1.84% nonbinary/other, with *M*_age_ = 36.91 and *SD*_age_ = 12.92. This study was granted exemption (IRB#1706865-2) by the University of California, Davis Institutional Review Board because it only included surveys for which ethical approval is not required. Informed consent was obtained electronically within the survey.

Preregistration, data, and study materials for this project can be found at https://osf.io/qm5sd/?view_only=21b9fbe77d9d44b1be85f92af736d436.

### Measures

We used the 20-item Mini International Personality Item Pool (Mini-IPIP; Donnellan et al., 2006) to score participants’ personality on a nine-point Likert-type scale ranging from 1 = “*Strongly disagree*” to 9 = “*Strongly agree*.” We first administered the Mini-IPIP with no reference group prompt to measure participants’ unprompted personality scores. Following the unprompted personality assessment, we readministered the Mini-IPIP six times with specific reference group prompts for others in general, close others, others the same age, others the same gender, past self, and ideal self. These instructions were given in the same order across participants. Thus, the respondents completed 140 questions (7 reference groups × 20 items). Internal consistency values for each trait across each reference group are given in Table 1.

**Table 1.** Internal Consistencies Across Conditions

Reference group condition	Extraversion		Agreeableness		Conscientiousness		Neuroticism		Openness	
	α	ω_*t*_	α	ω_*t*_	α	ω_*t*_	α	ω_*t*_	α	ω_*t*_
Unprompted	.87	.89	.83	.89	.78	.81	.78	.83	.80	.88
People in general	.85	.89	.84	.90	.80	.84	.79	.85	.81	.87
Close others	.82	.86	.81	.87	.79	.83	.75	.82	.77	.83
Same age	.84	.87	.81	.87	.77	.82	.73	.79	.77	.84
Same gender	.83	.86	.82	.87	.76	.82	.74	.81	.79	.85
Past self	.77	.83	.75	.83	.69	.75	.70	.78	.72	.80
Ideal self	.74	.82	.76	.84	.80	.86	.75	.81	.75	.79

### Analyses

We used repeated-measures analysis of variance models to test mean-level differences of each trait across reference group conditions. We used 95% confidence intervals to determine the significance of pairwise comparisons.

## Results

**Figure 1.**
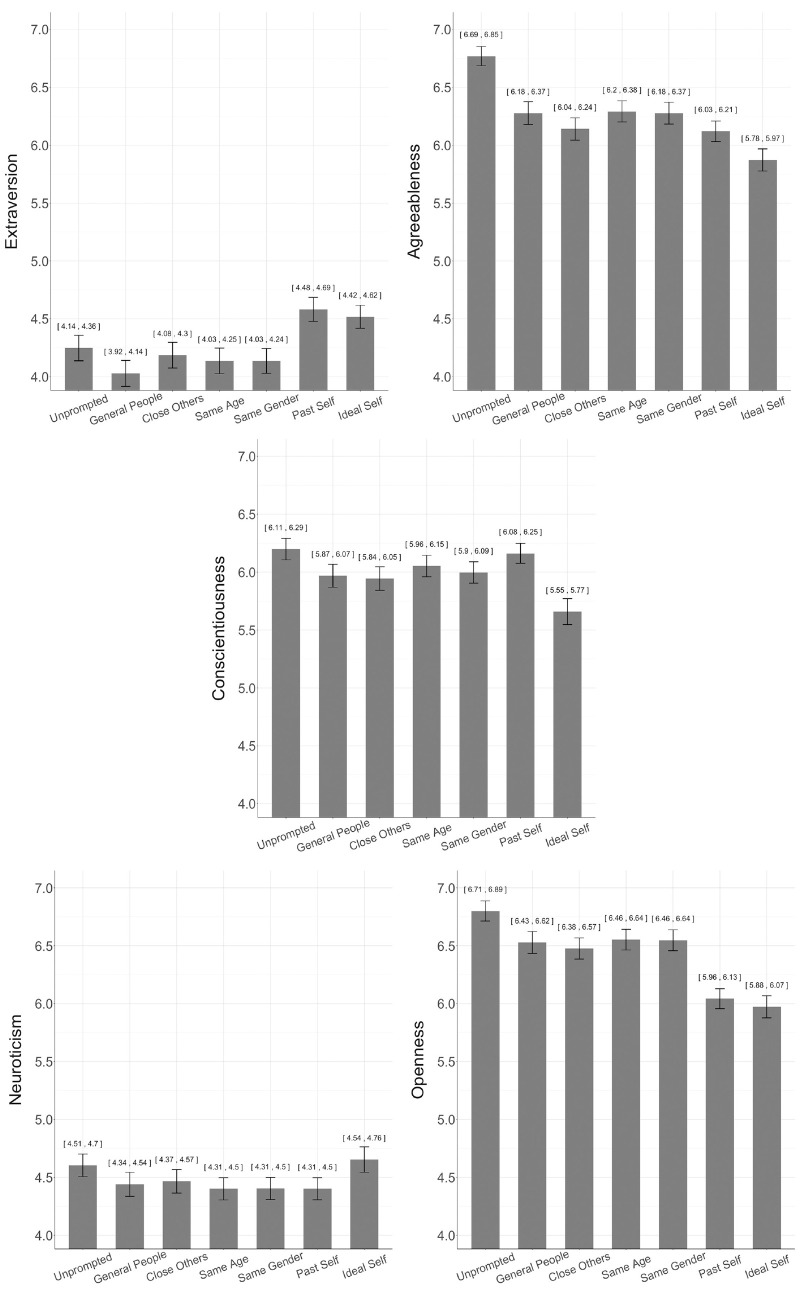
Mean Differences Between Reference Group Conditions Across Traits.

We observed significant differences across reference groups for all five traits (Table 2, Figure 1). We expected that the close others reference group (e.g., romantic partner, close friends/family) would produce lower conscientiousness scores than the other reference groups. With the exception that conscientiousness was lower for close others than for unprompted scores, this hypothesis was not supported by the results.

**Table 2.** Means and Standard Deviations Across Conditions

Reference group condition	Extraversion	Agreeableness	Conscientiousness	Neuroticism	Openness
	*M*(*SD*)	*M*(*SD*)	*M*(*SD*)	*M*(*SD*)	*M*(*SD*)
Unprompted	4.249 (1.944)	6.769 (1.475)	6.199 (1.635)	4.604 (1.722)	6.800 (1.527)
People in general	4.029 (1.975)	6.277 (1.727)	5.969 (1.741)	4.441 (1.834)	6.528 (1.663)
Close others	4.187 (1.958)	6.140 (1.698)	5.945 (1.784)	4.467 (1.782)	6.476 (1.614)
Same age	4.138 (1.947)	6.291 (1.597)	6.053 (1.655)	4.401 (1.685)	6.553 (1.571)
Same gender	4.137 (1.878)	6.276 (1.657)	5.997 (1.633)	4.404 (1.672)	6.548 (1.588)
Past self	4.582 (1.831)	6.120 (1.565)	6.162 (1.512)	4.402 (1.678)	6.043 (1.510)
Ideal self	4.518 (1.747)	5.873 (1.677)	5.659 (1.974)	4.654 (1.929)	5.972 (1.672)

We expected the ideal self reference group to produce more maladjusted levels of all Big Five traits, that is, lower levels of agreeableness and conscientiousness and higher levels of neuroticism. There was fairly consistent evidence for this hypothesis. The ideal self condition produced higher neuroticism scores than all but the unprompted condition and lower agreeableness and conscientiousness scores than all other conditions.

We expected that the past self reference group would produce more well-adjusted levels of the Big Five traits, that is, higher levels of agreeableness and conscientiousness and lower levels of neuroticism. Instead, the past self condition produced lower scores for agreeableness and similar scores compared with other conditions for neuroticism and conscientiousness.

**Figure 2.**
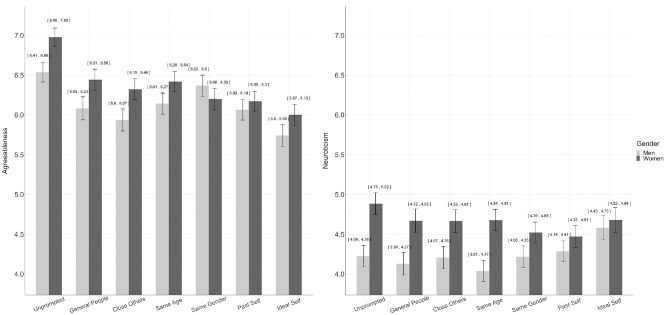
Neuroticism and Agreeableness Scores Separated by Gender.

We expected the same gender reference group for women to produce lower scores on agreeableness and neuroticism and higher scores on these traits for men than other reference group conditions. The only support for this hypothesis had to do with men’s agreeableness scores (Figure 2). Men had higher agreeableness scores in the same gender condition than in any condition other than the unprompted condition. Although women had higher agreeableness scores than men in all conditions other than same gender, women’s agreeableness scores were lower for the same gender comparisons than those for the unprompted condition, higher than those for the ideal self condition, and in the same range for all other conditions. There was no discernable interaction between gender and the same gender reference group for neuroticism. It is worth noting, however, that gender differences between men’s and women’s levels of agreeableness and neuroticism are the least contrasted values in the same gender, past self, and ideal self conditions, with same gender being the only between-person comparison, pointing to the weight the opposite gender carries when making trait comparisons.

## Discussion

The goal of this study was to examine how instructions for respondents to compare themselves to particular reference groups when completing a personality trait questionnaire affect personality trait scores. Several interesting patterns emerged. First, instructions to make within-person comparisons (i.e., to either the past or ideal self) seemed to have a larger impact on scores than instructions to make between-person comparisons (e.g., other people in general, to whom the respondent feels close, or who is the same in gender or similar in age) (Cohen’s *d* range .00-.98). Second, the effect of between-person reference group instructions tended to be very small (i.e., Cohen’s *d* < .05). Third, unprompted instructions consistently produce somewhat higher scores than between-person reference group instructions (Cohen’s *d* range .03-.40). Each of these effects will be discussed in turn.

### Within vs. Between-Person Comparisons

There is a qualitative difference between being asked to rate your personality relative to some group of people, however large, diverse, or clearly specified, and being asked to rate your personality in comparison to some other version of yourself. In this study, we asked respondents to compare themselves not only to groups of people but also to their past self and their ideal self. One of the most consistent patterns in the results was that there was a great deal of consistency between all the between-person instruction prompts, and furthermore, these prompts produced scores that were largely similar to responses without a prompt. This suggests that people seem to be making between-person comparisons when completing a questionnaire, as would be generally expected by clinicians and personality researchers and is believed by respondents themselves (Lenhausen et al., 2022).

In contrast, there was a fair amount of variability in results using the within-person prompts. As predicted, people tended to rate their personality traits as less adaptive—less agreeable, less conscientious, less open, and more neurotic—when comparing themselves to their own ideal than they do when comparing themselves to others. Interestingly, they also rated themselves as more extraverted when comparing themselves to their ideal self than they did when comparing themselves to others. There may be interesting applications to prompting people to compare themselves to their ideal self. For instance, C. R. Rogers and Dymond (1954) showed that the similarity between self-perceived real and ideal self served as a good proxy for psychological adjustment that tended to increase over the course of psychotherapy. However, the implication of these findings is that when these kinds of within-person comparisons are made, they are unlikely to be commensurate with or directly comparable to scores from standard personality measures.

An even larger and much less intuitive effect was observed when people were asked to compare their current self to their past self. Based on fairly well-established findings that personality tends to mature, meaning that agreeableness and conscientiousness tend to increase and neuroticism tends to decrease, during young and middle adulthood (Bleidorn, 2015; Schwaba et al., 2022), we expected participants in this sample to see themselves as more mature now than in the past. However, scores for the past self reference group instruction did not reflect this maturation as agreeableness and openness were lower and conscientiousness and neuroticism were rather unchanged compared with those for other conditions. This does not directly indicate that people see themselves as having become less agreeable and open over time, but it is suggestive of that kind of pattern. When coupled with previous findings from these data that people tend to believe that their unprompted scores would be most similar to scores from the others in general prompt, but actually their unprompted scores were most similar to others my same age prompt, these findings may have important implications for developmental research. In particular, age-graded comparisons across time could obscure developmental patterns, and people may not have perfect insight about how they have changed over time (Hopwood et al., 2022). However, it is important to keep in mind that these data were collected during the COVID-19 pandemic. Although evidence is mixed regarding the effects of COVID-19 on personality change (Bühler et al., 2021), it seems plausible that people perceived themselves as less agreeable and open during the pandemic, which is the pattern we observed. Moreover, we did not specify how far in the past the respondent should consider (e.g., 1 month, 1 year, 5 years), and it is possible that findings were affected by variation in which past self respondents had in mind.

Existing research that can speak to the level of insight people have about their own personality change offers a complex picture. Evidence from multiple studies indicates that people’s lay beliefs about how personality changes generally match the empirical finding that personality matures during development, and particularly during young adulthood (Haslam et al., 2007; D. Wood & Roberts, 2006). Gutral et al. (2022) found that people have different beliefs about how they have changed in the past relative to how they will change in the future although perceived changes tend to be positive when present. Hudson et al. (2021) found that beliefs about the magnitude and direction of personality change were largely unrelated to changes measured subsequently in a longitudinal design. However, Schwaba et al. (under review) found that people largely made correct inferences about how their subjective experience of past life events had changed their personality. Future longitudinal research should test these ideas by using specific reference group prompts, including questions about how people believe they have changed, in designs that capture normative change and change as a function of life experiences or interventions.

It is interesting that asking people to compare themselves to specific other groups of people did not impact trait scores all that much. Given well-known personality differences between men and women or across age, we expected people to have different scores depending on which group they were considering. This suggests that people may not have nuanced ideas about how subgroups differ in personality features or may not use that kind of information to inform how they rate themselves. At a practical level, it suggests relatively little benefit to prompting different specific reference groups, at least in terms of the average trait scores that would likely result.

### The Effect of Unprompted Reference Groups

However, an interesting and unexpected pattern emerged suggesting that people tended to have somewhat higher trait scores in an unprompted instruction set than in any of the between-person prompts. If we assume that people are generally making between-person comparisons when completing a personality questionnaire in the absence of specific reference group instructions, this may suggest that there is something about considering a specific reference group that tends to lower scores. Interestingly, this occurred across all traits, suggesting that it is not related to the valence or desirability of traits. Moreover, the specific reference group prompts included both the largest possible group (others in general) and specific subgroups (same age, gender), so the effect cannot be attributed to comparisons to particular kinds of groups. Instead, the pattern of results suggests that thinking about reference groups, in general, perhaps makes people more conservative in filling out questions about themselves.

### Implications for Applied Assessment

As discussed above, accounting for RGEs may be one way to reduce bias and increase validity in applied psychological assessment. The most direct way to do this would be to give specific instructions about which reference group the respondent should compare themselves to. These prompts may vary by setting. For instance, in situations when groups will be compared such as cross-cultural research, it might be beneficial to give all respondents the same reference group to homogenize RGEs and better isolate substantive group differences. In contrast, in longitudinal research focused on outcomes, it may be beneficial to focus respondents on how they are different relative to a past version of themselves. Some work shows that context can matter, such that framing the reference to specific settings, such as work or home, can increase assessment validity (Lievens et al., 2008). Specific reference group instructions are relatively uncommon on most assessment instruments, and the actual impacts of these instructions, such as how much respondents attend to them or use them accurately, remains an important question for future research. However, the results of the current study provide some useful information about what might be expected under different prompt conditions.

### Design Considerations

All these results should be considered preliminary given that this kind of research is relatively uncommon and that certain design features could have influenced results. Indeed, we based our hypotheses on results from previous studies (Credé et al., 2010), and although certain patterns did emerge, they were only somewhat consistent with what we predicted. Particular design features that could have influenced our results include our sampling strategy and population, the fact that we did not randomize conditions, our use of a particular personality instrument, and our use of particular instruction sets. Further work is needed to replicate these findings and extend them by systematically changing design features that could produce different outcomes. Moreover, this study was conducted in a North American convenience sample, and it is unclear how well results would generalize to other populations.

## Conclusion

These results and those of previous studies do suggest that personality scores vary as a function of who the respondent is comparing themselves to while completing items. At a basic level, these results suggest an important source of systematic variation in personality test scores that is largely unrelated to the traits being measured, which should be considered when interpreting results. This is especially the case when making comparisons across groups (e.g., cultural groups) or across time. At a more applied level, these results point to the need to carefully consider the value of specific reference group prompts, ideally based on empirical findings regarding the impact of those kinds of prompts. This study provides some evidence regarding the impacts that might be anticipated, but given the discrepancy across studies, they also show that more work is needed in this area before firm conclusions can be drawn.
